# Scalable Preparation of Low-Defect Graphene by Urea-Assisted Liquid-Phase Shear Exfoliation of Graphite and Its Application in Doxorubicin Analysis

**DOI:** 10.3390/nano10020267

**Published:** 2020-02-05

**Authors:** Chang-Seuk Lee, Su Jin Shim, Tae Hyun Kim

**Affiliations:** Department of Chemistry, Soonchunhyang University, Asan 31538, Korea; eriklee0329@sch.ac.kr (C.-S.L.); sujean0395@naver.com (S.J.S.)

**Keywords:** graphene, mass production, shear exfoliation, physical exfoliation

## Abstract

The mass production of graphene is of great interest for commercialization and industrial applications. Here, we demonstrate that high-quality graphene nanosheets can be produced in large quantities by liquid-phase shear exfoliation under ambient conditions in organic solvents, such as 1-methyl-2-pyrrolidinone (NMP), with the assistance of urea as a stabilizer. We can achieve low-defect graphene (LDG) using this approach, which is relatively simple and easily available, thereby rendering it to be an efficient route for the mass production of graphene. We also demonstrate the electrochemical sensing of an LDG-modified electrode for the determination of doxorubicin (DOX). The sensor shows an enhanced electrocatalytic property towards DOX, leading to a high sensitivity (7.23 × 10^−1^ μM/μA) with a detection limit of 39.3 nM (*S/N* = 3).

## 1. Introduction

Graphene can be applied to everything from electronics and pharmaceuticals to sensing, biomedical and energy devices [1,2]. With exceptional properties derived from the atom-thick layer of graphite, it is becoming more prevalent and more critical in industry and scientific research. Although there is a myriad of uses for this wonder material, production can often be expensive and time-consuming. Graphene can be prepared using epitaxial growth and chemical vapor deposition (CVD) [3,4]. Even with the production of high-quality graphene, however, these techniques suffer from the drawbacks of complicated procedures, the need for toxic chemicals, expensive production or limited quantity, making it difficult to scale up to mass production for commercialization and industrial-scale application. With respect to mass production, chemical exfoliation is the facile and cost-effective technique in which the graphene preparation begins with the oxidation of graphite to graphene oxide (GO) and its subsequent reduction to get reduced graphene oxide (rGO) [5,6]. However, the resulting rGO is low-quality graphene with many of the defects of oxygen-containing functional groups.

To circumvent these issues, one of the most promising techniques is the physical exfoliation of layered graphite to graphene, which can lead to the mass production of low-defect graphene (LDG) [7]. The mechanical forces in physical exfoliation, such as sonication [8,9], ball milling [10,11] and shear exfoliation [12,13] can overcome the van der Waals forces that hold the graphite layers together, enabling graphene production on a large scale at a low cost. Graphene prepared from sonication and the ball-milling method, however, has more fragmentation and defects than expected, which is attributed to the sonication-induced cavitation, or collisions among the grinding media during the milling process. On the other hand, liquid-phase shear exfoliation with a high-shear mixer is easily available and makes it relatively simple to mass-produce defectless graphene. The size and amount of graphene produced through shear exfoliation can be controlled by the shearing speed, time, pore size, diameter of the rotor, stabilizers, and species of solvent [13]. In particular, the stabilizer facilitates the exfoliation of graphite to create a single layer of graphene by penetrating into the edges of the graphite layers, acting as ‘‘molecular wedge” and preventing the self-aggregation of the graphene in water through the intersheet steric and/or electrostatic repulsion force [14,15,16,17]. However, the surface residuals of stabilizers on graphene surfaces can seriously decrease the extraordinary electrical performance. Thus, the removal of stabilizers from graphene sheets is necessary to utilize graphene in practical applications. Recently, various chemicals have been introduced as a stabilizer for liquid-phase exfoliation, due to their easy-to-remove and easy-to-control qualities, low cost, and safety [16,17]. However, there still remains a challenge to improve efficiency for the scalable preparation of graphene. Meanwhile, species of solvent are also one of the key factors for enhancing the production efficency and dispersion homogeniety of graphene. A variety of organic solvents have been utilized for the exfoliation studies. The most common solvents are isopropyl alcohol (IPA) [18], N,N-dimethylformamide (DMF) [19], dimethyl sulfoxide (DMSO) [20], 1-methyl-2-pyrrolidinone (NMP) [21], etc., because they can minimize the interfacial tension with graphene due to their surface tension being similar to that of graphene.

In this study, we demonstrated that graphite can be exfoliated to give large quantities of graphene nanosheets by the liquid-phase shear exfoliation method, using a homogenizer in NMP as an organic solvent, with the assistance of urea as a stabilizer, under ambient conditions. Remarkably, we could obtain non-oxydized LDG sheets on a large scale under optimal conditions. Furthermore, the LDG was used to modify an electrode for fabricating the electrochemical sensor of doxorubicin (DOX). This sensor showed a good sensitivity and selelctivity for the electrochemical detection of DOX. This approach has a great potential in the industrial-scale production of graphene with good quality for practical applications.

## 2. Experimental

### 2.1. Chemicals and Reagents

The chemicals and materials were purchased from Sigma-Aldrich, Inc. (St. Louis, MO, USA), as follows: natural graphite powder, NMP, phosphate buffer saline (10 mM, pH 7.4), glucose, KCl, NaCl, urea, doxorubicin HCl, tryptophan and commercial graphene. The deionized (DI) water was prepared using water purification systems (specific resistivity > 18 MΩ cm, MilliQ, Millipore Korea, Co., Ltd., aqua Max Ultra 370 (Younglin Instrument Co., Anyang, Kyounggi-do, Korea).

### 2.2. Instrumentation and Measurements

The shear exfoliation was performed with an L5M-A mixer homogenizer (Silverson, Co., East Longmeadow, MA, USA) with a Square Hole High Shear Screen^™^ workhead. The absorbance of the exfoliated LDG was measured by UV-vis spectroscopy (SINCO Co., Ltd., Seoul, Korea). The concentrations of the LDG were calculated from the standard curve for the absorbance at 660 nm of the commercial graphene solution. The Raman spectroscopy was performed using an EnSpectr R532 Raman spectrometer (Enhanced Spectrometry, Inc., San Jose, CA, USA). Transmission electron microscopy (TEM) was carried out on a JEOL 2010 device (JEOL, Inc., Peabody, MA, USA) with an accelerating voltage 200 keV. The X-Ray diffraction (XRD) patterns were recorded using a Rigaku SmartLab X-ray diffractometer (Rigaku Co., Tokyo, Japan) with Cu Kα source. Fourier transform infrared spectroscopy (FTIR) was performed with a NICOLET iS10 (Thermo Scientific Korea Ltd., Seoul, Korea). The X-ray photoelectron spectroscopy (XPS) signals were recorded using a Thermo Scientific K-Alpha XPS system (Thermo Fisher Scientific, Paisley, UK) equipped with a micro-focused monochromatic Al Kα X-ray source (1486.6 eV). The electrochemical measurements were performed with a CHI 660D electrochemical workstation (CH Instruments, Inc., Austin, TX, USA). A conventional three-electrode cell was used with a Pt wire as a counter electrode, an Ag/AgCl electrode was used as a reference electrode and a bare glassy carbon electrode (GCE, diameter = 3 mm), or modified electrode, was used as a as working electrode at room temperature.

### 2.3. Shear Exfoliation of Graphite by Using a Homogenizer

**Scheme 1:**
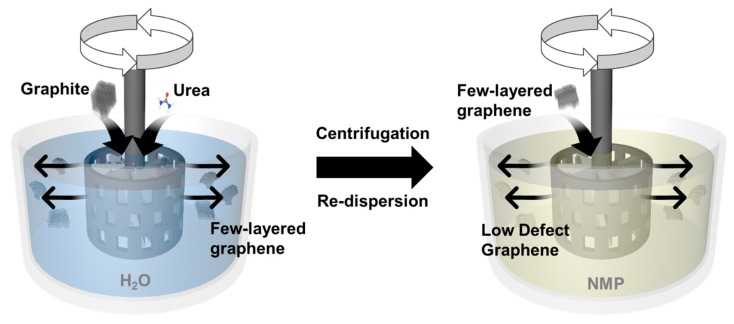
Illustration of mass production of low-defect graphene (LDG) by the urea-assisted liquid-phase shear exfoliation of graphite.

The LDG samples were prepared by the liquid-phase shear exfoliation method using a high-speed mixer homogenizer, as shown in Scheme 1. In a typical procedure, 1 g graphite was added into the 100 mL of aqueous urea solution (100 g/100 mL). The mixture solution was sonicated in a sonication bath (4 W) for 20 min at room temperature to dissolve the urea. After sonication, the mixture solution was exfoliated by the mixer homogenizer at a speed of 3000 rpm for 4 h. Then, to remove the urea, the exfoliated few-layered graphene was collected by centrifugation at 3000 rpm for 20 min, twice. The pellet of few-layered graphene was re-dispersed in 100 mL NMP. The resultant dispersion was mixed by the mixer homogenizer at a speed of 3000 rpm for 5 h. Finally, to obtain the exfoliated LDG, the mixture solution was centrifuged at 3000 rpm for 20 min and the supernatant was collected for the LDG solution. The LDG solution was evaporated under a vacuum to discard the solvent at 50 °C.

### 2.4. Preparation of Graphene Electrode for Electrochemical Sensor

Before the electrode fabrication, the GCE was polished with 1.0, 0.3, and 0.05 μm Al_2_O_3_ powder and washed in an ultrasonic bath for 10 min, then rinsed with DI water thoroughly. After that, 5 μL of 1 mg/mL LDG in ethanol was drop-casted onto the GCE and dried with an infrared spectroscopy (IR) lamp for a few minutes, then rinsed with DI water several times. Before the electrochemical sensing experiments, the LDG-modified GCE was activated electrochemically by cyclic voltammetry (CV) in 10 mM PBS buffer solution (pH 7.4); the potential was scanned from 0 to 1.0 V (vs. Ag/AgCl) three times with a 5 mV/s scan rate.

## 3. Results and Discussion

### 3.1. Effect of Processing Parameters on Graphene Concentration

**Figure 1:**
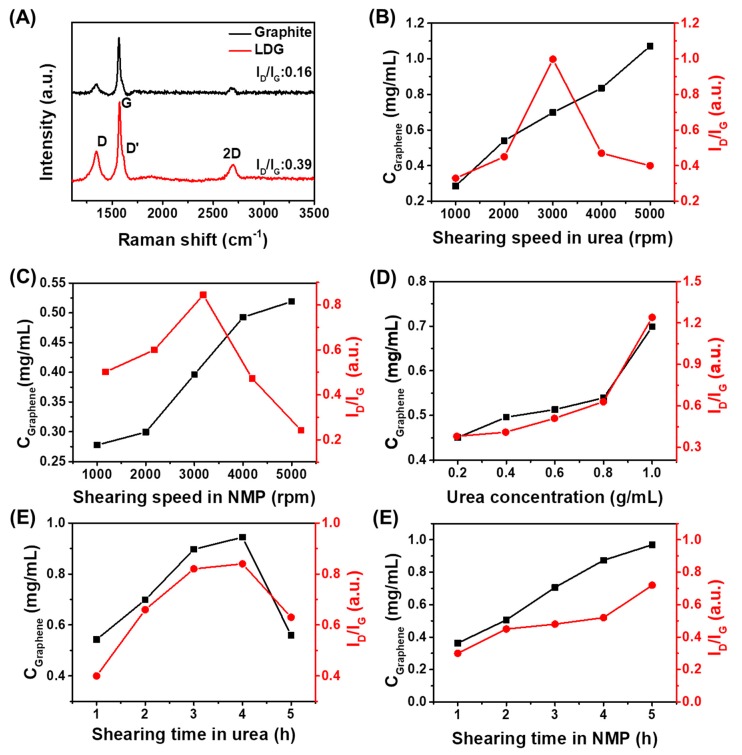
(**A**) Characterization of LDG by Raman spectroscopy. The influences of the processing parameters on the concentration (Left *y*-axis) and the I_D_/I_G_ ratio in Raman spectroscopy of the LDG solutions (Right *y*-axis). (**B**) The shearing speed in urea, (**C**) the shearing speed in NMP, (**D**) the urea concentration, (**E**) the shearing time in urea, and (**F**) the shearing time in NMP.

To confirm the production of the LDG sheets, as described in Scheme 1, Raman spectroscopy was first employed to monitor the exfoliation of graphite into graphene sheets. As shown in Figure 1A, three peaks were observed at 1339 (D band), 1573 (G band) and 2688 cm^−1^ (2D band) for both graphite and LDG. Using the ratio of peak intensities I_D_/I_G_ in Raman spectra, the level of disorder in graphene can be characterized. As the disorder in the graphene increases, *I_D_/I_G_* displays two different behaviors, as follows: (i) at a low-defect concentration, like pure graphite materials, *I_D_/I_G_ ≃ 1/L_d_^2^* (Stage 1); (ii) at a relatively high defect concentration, like produced graphene by chemical or physical exfoliation, *I_D_/I_G_ ≃ L_d_^2^* (Stage 2), where *L_d_* represents the mean distance between two defects [22,23]. The transition between Stage 1 and 2 is usually observed at *I_D_/I_G_* ≃ 3 [22]. So, in the present case, the *I_D_/I_G_* value increased as the level of defects decreased. The exfoliation caused an increase in the I_D_/I_G_ value to 0.39 in the LDG, which is higher than that of graphite (0.16), but considerably lower than that of GO reported previously [24]. This should be attributed to the edge defects of the exfoliated samples rather than the lattice destruction, meaning that very few defects existed in the as-prepared LDG [25]. In addition, the 2D band of LDG turned out to be a relatively symmetrical shape and appeared at lower position (~10 cm^−1^) when compared to the 2D band of graphite. A shoulder peak on G band, along with the D, G and 2D band, appeared at 1616 cm^−1^ in the LDG, which is namely D band and is evidence of few-layer graphene. These results successfully demonstrate the reduction in the number of graphene layers and the subsequent formation of few-layer graphene with a low density of defects [26].

To optimize the preparation procedure, we investigated the effects of the processing parameters, such as the shearing speed, urea concentration, shearing time in urea and shearing time in NMP on the concentration of LDG solutions and the intensity ratio of the D band and G band (*I_D_/I_G_*) using Raman spectroscopy (Figure 1B–E). The processing parameters were optimized with the maximal concentration and the highest *I_D_/I_G_* value of LDG, indicating the lowest level of defects. To test the influence of the processing parameters, only one parameter was varied, while the other parameters were kept constant. Figure 1B shows the effect of the shearing speed in the urea solution on the concentration and the *I_D_/I_G_* value of LDG dispersions. As the shearing speed increased in the range of 1000 to 5000 rpm, the concentration of LDG also increased. However, the *I_D_/I_G_* of LDG dispersions reached its maximum at 3000 rpm and decreased after that. This indicates that the highest LDG concentration with the lowest level of defects was obtained at a shearing speed of 3000 rpm. Therefore, 3000 rpm was selected as the optimal shearing speed for the preparation of LDG dispersion. A similar trend was observed in the test for the effect of the shearing speed in NMP, showing the highest *I_D_/I_G_* value of LDG at 3000 rpm, although the concentration increased with increasing shearing speed (Figure 1C). This is because the graphene size was reduced with the increase of edge defects and agglomeration, as the shear force increased with the shearing speed. Thus, 3000 rpm in NMP was enough to achieve a high yield of good quality. The effect of the urea concentration on the exfoliation efficiency of graphite was also examined (Figure 1D). The concentration and the *I_D_/I_G_* value of LDG dispersions increased with increasing concentrations of urea. This result suggests that the urea molecules could expand the interlayer space of graphite to overcome the van der Waals force between graphite layers and, thus, LDG concentration significantly increased due to the presence and adsorption of more concentrated urea molecules. A suitable concentration of urea was chosen to be 100 g/100 mL, due to the solubility of urea (107.9 g/100 mL in 20 °C). The effect of the shearing times in aqueous urea solution and NMP, respectively, were further evaluated in the range of 1 to 5 h (Figure 1E,F). The highest concentrations and I_D_/I_G_ values of LDG dispersions were achieved at 4 h in urea and 5 h in NMP and, thus, they were chosen as the optimal shearing times. Interestingly, the graphene prepared without urea assistance showed a lower concentration and I_D_/I_G_ value than those of LDG (Figure S1, Supplementary Materials), proving the role of urea as a stabilizer and a molecular wedge in shear exfoliation. Taken together, these results show that the highest concentration of LDG with high quality can be prepared under the following processing parameters: (i) the shearing speed in urea solution is 3000 rpm; (ii) the shearing speed in NMP is 3000 rpm; (iii) the urea concentration is 100 g/100 mL; (iv) the shearing time in urea is 4 h; (v) the shearing time in NMP is 5 h.

### 3.2. Characterization of Low-Defect Graphene

**Figure 2:**
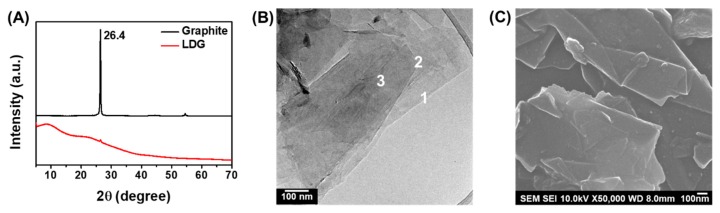
Characterization of LDG by (**A**) X-ray diffraction spectroscopy, (**B**) TEM, and (**C**) SEM.

The XRD patterns of the LDG and graphite powder were utilized to confirm the exfoliation of the graphite to graphene sheets (Figure 2A). While the XRD pattern of the graphite showed a sharp diffraction peak at 2*θ* = 26.5°, corresponding to a d-spacing of 0.34 nm, the LDG showed a broad peak around 24° and very weak diffraction peak around 26.5°, which indicates the exfoliation of graphite. The LDG also exhibited a broad peak around 10°, demonstrating that the interlayer spacing was expanded during the exfoliation [27,28]. As shown in Figure 2B,C, both TEM and SEM images also revealed the microstructures of the exfoliated graphene sheets in the LDG dispersions. The reduction in the thickness of the graphene sheets (mono-, bi- and tri-layer, Figure 2B) and their lateral size (~1000 nm) is evident compared with the graphite flakes, confirming the high degree of exfoliation of the process. The SEM image of the LDG clearly shows a thin planar structure with curled and irregular edges (Figure 2C). According to statistical analysis based on the SEM and TEM images of the LDG sheets, lateral size and layer distribution were measured in Figure S2. By measuring ~90 graphene sheets, a lateral size histogram (Figure S2A) displays that more than 84% of the graphene sheets are larger than 3 μm, which is much higher than that of liquid phase exfoliated graphene sheets by sonication [14,29]. The layer distribution (Figure S2B) shows that monolayer graphene accounts for 48.8% of the graphene sheets in the dispersion. We believe that the large sheet size and high exfoliation efficiency are related to the intercalation of urea molecules into the graphite and the liquid phase shear exfoliation process. These results suggest the successful preparation of well-exfoliated and non-oxidized graphene sheets with high quality, verifying that this approach is a nondestructive and efficient method to produce LDG sheets on a large scale.

**Figure 3:**
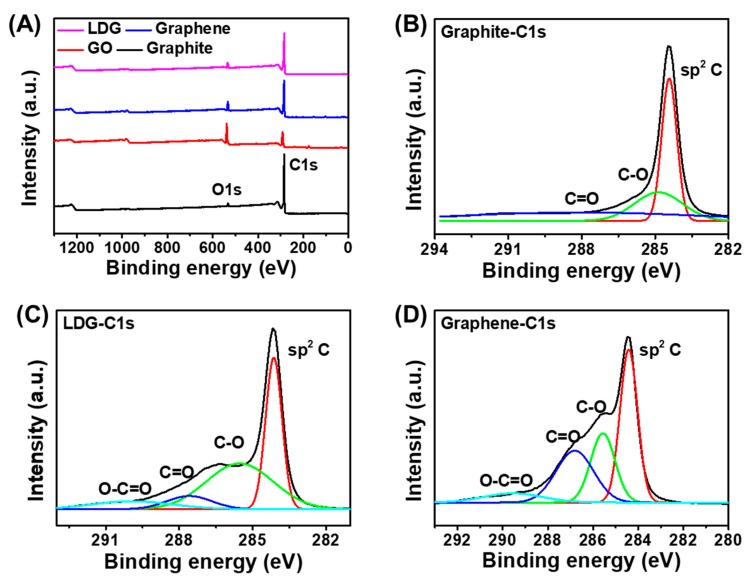
(**A**) XPS survey of LDG powder, commercially available graphene, graphene oxide and graphite with high-resolution C 1s spectra of (**B**) graphite, (**C**) LDG, and (**D**) commercially available graphene, respectively.

XPS was performed to probe the chemical composition and binding states of the exfoliated LDG (Figure 3, Figure S3 and Table S1). The XPS survey curves of LDG, commercially available graphene and graphite show a pronounced peak at 285 eV and a weak peak at 533 eV, except for graphene oxide (GO), which displayed considerable peak heights at both 292 and 538 eV, due to the oxygen functionalities derived from chemical exfoliation (Figure 3A). Based on the results from XPS, the oxygen content of the LDG powder was estimated to be 6.4 atomic %, thus being higher than that of the graphite precursor (3.0%), but considerably lower than that of GO (32.5%) and even that of commercially available graphene (11.1%), as summarized in Table S1. The overall oxygen of LDG is associated with the edge defects of LDG in the form of oxide groups, which is in agreement with the results from Raman and XRD spectra, and consistent with the aforementioned higher crystalline size of graphene sheets [30]. The calculated C/O ratio of the LDG (14.6) is significantly higher than that of commercially available graphene (8.0) and that of GO (2.1). Figure 3B–D show the C 1s core-level spectra of graphite, LDG, and commercially available graphene, respectively. The pattern of LDG (Figure 3C) exhibits a similar structure to that of graphite (Figure 3B), revealing that the intrinsic in-plane crystal structure of graphite remains intact during the shear exfoliation treatment. The XPS data of the C 1s profiles also prove that the LDG (Figure 3C has fewer oxygen-containing functional groups (such as C-O, C=O, and COO-) than GO (Figure S1) and even commercially available graphene (Figure 3D).

### 3.3. Electrochemical Detection of Doxorubicin with LDG Electrode

To assess the performance of LDG as a sensing material for practical applications, we fabricated the LDG-modified GCE (LDG-GCE) for the detection of DOX. DOX, as one of the clinically important anti-cancer drugs, has been extensively used in the treatment of various cancers. However, long-term clinical use of DOX is limited due to its side effects, such as systemic toxicity, drug resistance, and life-threatening heart damage. Therefore, the development of highly sensitive analytical techniques for DOX detection is of great significance in clinics and pharmaceutics.

**Figure 4:**
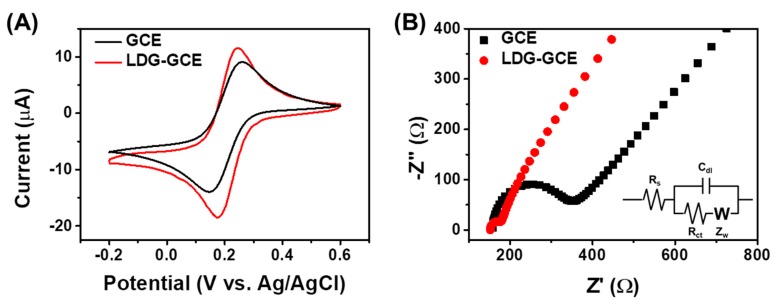
(**A**) CV curves of GCE (black) and LDG-GCE (red) in 0.1 M KCl containing 3 mM of K_3_Fe(CN)_6_. Scan rate: 50 mV/s. (**B**) The Nyquist plots of the GCE (black) and LDW-GCE (red) in 0.1 M KCl containing 3 mM of K_3_Fe(CN)_6_, using a frequency range of 0.1 to 10^5^ Hz, a bias potential of +0.214 V and an AC amplitude of 10 mV. (Inset: Randles equivalent circuit model).

Before the electrochemical sensing experiments, CV and electrochemical impedance spectroscopy (EIS) were performed to investigate the charge transfer ability of LDG-GCE in 0.1 M KCl solution containing 3 mM of K_3_Fe(CN)_6_. As shown in Figure 4A, both LDG-GCE and GCE show a pair of well-defined redox peaks. However, the peak potential difference (*ΔE_p_* = 70 mV) of LDG-GCE is smaller than that of bare GCE (119 mV), while the peak currents for LDG-GCE are significantly higher, indicating that the LDG-GCE electrode exhibited a better electrochemical performance. The EIS experiments also showed better electron-transfer characteristics for LDG-GCE. The charge transfer resistance (*R_ct_*) of LDG-GCE was estimated to be 25.9 Ω from the diameter of a semicircle in the high frequency region of a Nyquist plot (Figure 4B), which was much smaller than that of the GCE (*R_ct_* = 168.8 Ω). This suggests that rapid electron transfer occurred on the LDG-GCE surface.

**Figure 5:**
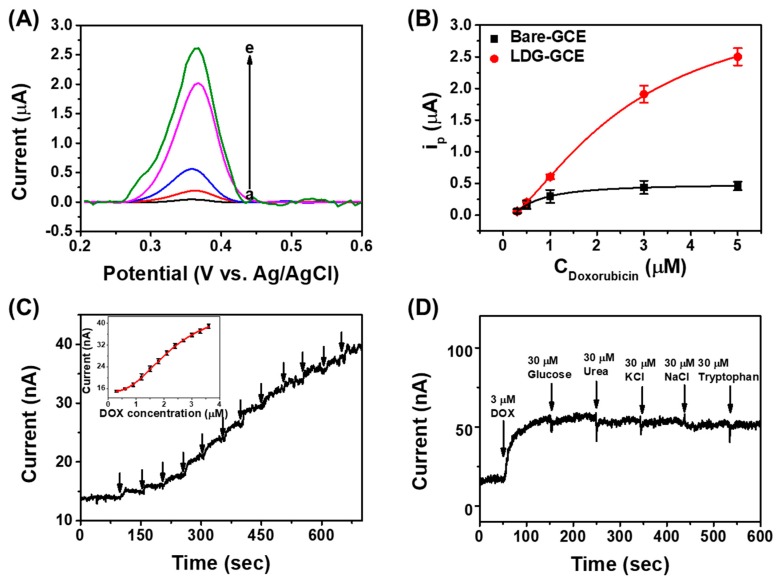
(**A**) Differential pulse voltammetry (DPV) curves of LDG-GCE in 10 mM PBS solution (pH = 7.4) containing various concentration of DOX (0.3, 0.5, 1.0, 3.0 and 5.0 μM. (**B**) The calibration plots of i_p_ vs. concentration of DOX from DPV at LDG-GCE and GCE, respectively. The average values and standard deviations were obtained based on three results. (**C**) The amperometric responses of LDG-GCE to the successive addition of DOX from 0.3 μM to 3.6 μM at 0.366 V vs. Ag/AgCl. (Inset: The calibration plot from the amperometric measurements) (**D**) The amperometric responses of LDG-GCE to the successive addition of 3 µM DOX, 30 µM glucose, 30 µM urea, 30 µM KCl, 30 µM NaCl, and 30 µM tryptophan. at 0.366 V vs. Ag/AgCl.

The electrochemical detection of DOX was performed with differential pulse voltammetry (DPV) and chronoamperometry in 10 mM PBS solution (pH = 7.4) containing various concentrations of DOX, using LDG-GCE. Figure 5A shows the typical DPV curves in which only one broad peak was observed at 0.366 V. Upon the addition of DOX in the range of 0.3 to 5.0 μM, the LDG-GCE exhibited an increase in the oxidation peak currents. The corresponding calibration plots for DOX showed a good linear relationship between the DPV peak currents and the concentration of DOX in the range of 0.3–3.0 μM. As is to be expected from the electrochemical performance of LDG-GCE, the sensor shows better sensitivity when compared with bare GCE (Figure 5B). The calculated sensitivity for the electrochemical detection of DOX was 7.23 × 10^−1^ μM/μA with LDG-GCE, which is higher than bare GCE (3.54 × 10^−1^ μM/μA). Moreover, the limit of detection (LOD) for LDG- GCE (39.3 nM) at an S/N ratio of 3 was also much better than that of bare GCE with LDG-GCE (254 nM) and bare GCE. These results suggest that the LDG-GCE exhibited an excellent performance with respect to the detection of DOX. To evaluate the practical applications in DOX analysis, chronoamperometric measurements were carried out in 0.1 M PBS (pH 7.4). Figure 5C shows the amperometric current–time (i–t) plots of the LDG-GCE at 0.366 V in response to the successive addition of DOX the PBS solution. The corresponding calibration curves are displayed as *i* (nA) = 1.06 × 10^−8^ + 0.00828 C (μM) in Figure 5C inset. The relationship between DOX concentration and the corresponding current is a linear one in the range of 0.3–2.7 μM with a LOD of 653 nM (*S/N* = 3). Table S2 summarizes and compares the analytical parameters of the LDG-GCE sensor with other electrodes, revealing that the proposed sensor shows a high sensitivity with a low LOD and suitable linear range. The selectivity experiments were performed by comparing the amperometric responses to DOX with potential interfering compounds found in biological fluids, such as glucose, urea, tryptophan, KCl, and NaCl. As shown in Figure 5D, no significant interference was observed during the amperometric detection of 3 µM DOX in the presence of 30 µM glucose, urea, KCl, NaCl, and tryptophan. This result underlines the suitable selectivity of LDG-GCE.

## 4. Conclusions

We have demonstrated the scalable production of graphene sheets with high quality by a simple and facile method, via NMP-mediated shear exfoliation of graphite with the assistance of urea. The successful production of LDG on a large scale was possible using a high speed mixer homogenizer by optimizing the processing parameters for the shear exfoliation, such as shearing speed, urea concentration, and shearing time by comparing the produced graphene concentration and I_D_/I_G_ value from the Raman spectra. The characterization experiments confirmed that the graphene sheets, produced by shear exfoliation with optimized parameters, are single layered, or few-layered at least, with negligible defects. In addition, using the LDG as a modification material for an electrode, we have fabricated an LDG-GCE electrochemical sensor for DOX, demonstrating sensitive and selective DOX analysis. Therefore, the proposed approach is promising for the industrial production of graphene for numerous applications.

## Supplementary Materials

The following are available online at https://www.mdpi.com/2079-4991/10/2/267/s1, Figure S1: Comparison of (A) the concentration and (B) the *I_D_/I_G_* ratio in Raman spectroscopy in graphene solution prepared with (black square dot) and without (red square dot) assistance of urea., Figure S2: (A) lateral size and (B) layer distribution of LDG calculated from 95 isolated sheets in SEM and TEM images., Figure S3: High resolution C1s spectra of GO., Table S1: XPS data of graphene oxide (GO), graphite, commercially available graphene and LDG., Table S2: Comparison of the analytical performance of LDG-GCE with other electrodes for DOX detection.
