# Supplementary material for: Scalable Preparation of Low-Defect Graphene by Urea-Assisted Liquid-Phase Shear Exfoliation of Graphite and Its Application in Doxorubicin Analysis
Source: Nanomaterials (Basel). 2020 Feb 5;10(2):267. doi: 10.3390/nano10020267 (PMC7075141; doi:10.3390/nano10020267)
Supplement: Supplementary file 1 [file nanomaterials-10-00267-s001.pdf]

nanomaterials

MDPI

Supporting information for

Scalable preparation of low defect graphene by urea-assisted liquid-phase shear exfoliation of graphite and its application in doxorubicin analysis

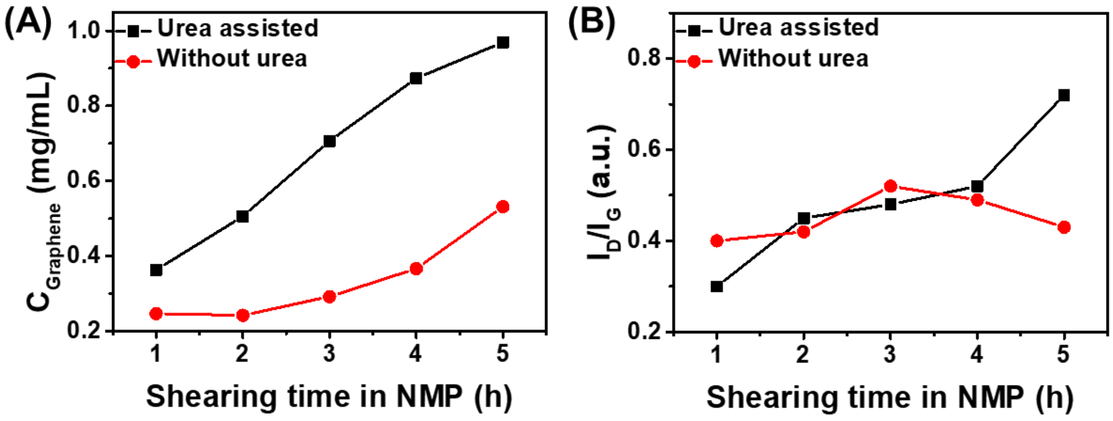

Figure S1. Comparison of (A) the concentration and (B) the I_D/I_G ratio in Raman spectroscopy in graphene solution prepared with (black square dot) and without (red square dot) assistance of urea.

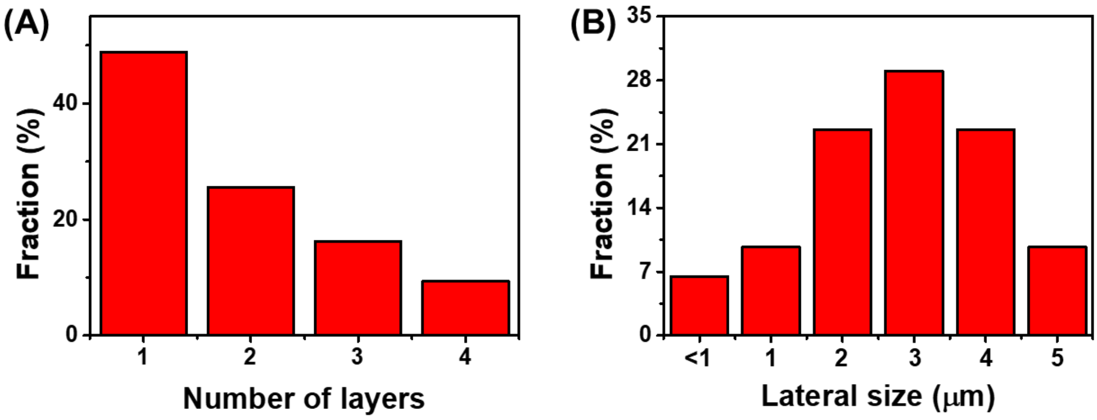

Figure S2. (A) lateral size and (B) layer distribution of LDG calculated from 95 isolated sheets in SEM and TEM images.

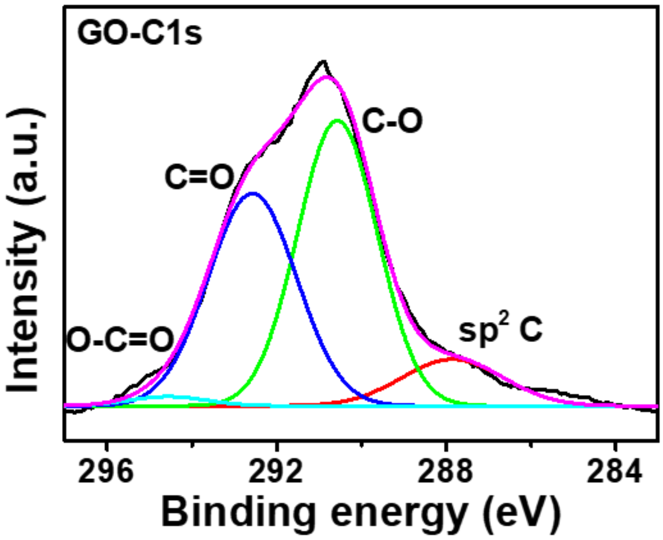

Figure S3. high resolution C1s spectra of GO.

Table S1. XPS data of graphene oxide (GO), graphite, commercially available graphene and LDG.

Sample	Element (atomic %)		
	C	O	C/O
GO	67.5	32.5	2.1
Graphite	97.0	3.0	32.2
Graphene	88.9	11.1	8.0
LDG	93.6	6.4	14.6

Table S2. Comparison of the analytical performance of LDG-GCE with other electrodes for DOX detection.

**Electrode**	**Method**	**Linear range (μM)**	**LOD (μM)**	**Reference**
SMDE	SWV	0.5 ~ 10.0	0.1	[1]
DRN-Apt/AuE	EIS	0.031 ~ 0.125	0.028	[2]
BPPDNi/Pt:Co-NPs/CPE	DPV	0.5 ~ 300	0.1	[3]
Carbon paste electrode	DCV and DPV	-	0.8 (DCV)<br>0.06 (DPV)	[4]
LDG-GCE	DPV and amperometry	0.3 ~ 3 (DPV)<br>0.3 ~ 2.7 (amperometry)	0.0393 (DPV)<br>0.653 (amperometry)	This work

1. Hahn, Y.; Lee, H.Y. Electrochemical behavior and square wave voltammetric determination of doxorubicin hydrochloride. *Arch. Pharm. Res.* **2004**, *27*, 31–34.
2. Bahner, N.; Reich, P.; Frense, D.; Menger, M.; Schieke, K.; Beckmann, D. An aptamer-based biosensor for detection of doxorubicin by electrochemical impedance spectroscopy. *Anal. Bioanal. Chem.* **2018**, *410*, 1453–1462.

3. Jahandari, S.; Taher, M.A.; Karimi-Maleh, H.; Mansouri, G. Simultaneous voltammetric determination of glutathione, doxorubicin and tyrosine based on the electrocatalytic effect of a nickel(II) complex and of Pt:Co nanoparticles as a conductive mediator. *Microchim. Acta* **2019**, *186*, 493.
4. Gooding, J.J.; Wasiowych, C.; Barnett, D.; Hibbert, D.B.; Barisci, J.N.; Wallace, G.G. Electrochemical modulation of antigen–antibody binding. *Biosens. Bioelectron.* **2004**, *20*, 260–268.
